# The *Varroa* paradox: infestation levels and hygienic behavior in feral *scutellata*-hybrid and managed *Apis mellifera ligustica* honey bees

**DOI:** 10.1038/s41598-023-51071-7

**Published:** 2024-01-11

**Authors:** Brandon Mukogawa, James C. Nieh

**Affiliations:** Department of Ecology, Behavior, and Evolution, School of Biological Sciences, University of California San Diego, 9500 Gilman Dr. MC 0116, La Jolla, CA 92093 USA

**Keywords:** Behavioural ecology, Animal behaviour

## Abstract

The *Varroa destructor* mite is a parasitic threat to managed and feral honey bee colonies around the world. Beekeepers use miticides to eliminate *Varroa* in commercial hives, but these chemicals can diminish bee health and increase miticide resistance. In contrast, feral honey bees have developed multiple ways to counteract mites without chemical treatment. We compared mite levels, grooming habits, and mite-biting behavior between feral Africanized honey bees (genomically verified *Apis mellifera scutellata* hybrids) and managed Italian honey bees (*A. mellifera ligustica*). Surprisingly, there was no difference in mite infestation levels between *scutellata*-hybrids and managed bees over one year despite the regular use of miticides in managed colonies. We also found no differences in the social immunity responses of the two groups, as measured by their hygienic habits (through worker brood pin-kill assays), self-grooming, and mite-biting behavior. However, we provide the first report that both *scutellata*-hybrids and managed honey bees bite off mite chemosensory forelegs, which the mites use to locate brood cells for reproduction, to a significantly greater degree than other legs (a twofold greater reduction in foreleg length relative to the most anterior legs). Such biting may impair mite reproduction.

## Introduction

The Western honey bee (*Apis mellifera*) plays a crucial ecological role as a pollinator in natural landscapes and agricultural ecosystems, contributing to about 13% of recorded flower visits worldwide^1,2^. *Apis mellifera* is the most prevalent pollinator in natural habitats and provides billions of dollars through its pollinator services in the United States alone^3^. However, honey bee colonies have been experiencing significant declines since 2006, with about one in three colonies dying annually, particularly in winter, from a range of factors that include pathogens, pesticide exposure, and nutritional deficiencies^4^. The most significant threat to honey bee colonies is *Varroa destructor*, a parasitic mite that feeds on honey bee fat reserves, causing reduced honey bee health and survival^5^. *Varroa destructor* is a parasite that requires honey bee hosts to survive and reproduce. In the phoretic phase, female mites attach themselves to adult honey bees and can thereby disperse to new colonies. Within the nest, the mites use chemosensory organs to help find brood cells in which they reproduce while feeding off the fat bodies and hemolymph of the brood^5,6^ .

The introduction of *Varroa* mites led to historic declines in both managed and feral honey bee colonies in North America^7,8^. Mite populations can grow quickly and managed honey bee colonies typically die within 2–3 years in the absence of effective control measures^6^. These mites have developed a growing resistance to miticides^9,10^, which adversely affect honey bee health^11,12^. *Varroa* mites also vector pathogens, such as the deformed wing virus (DWV), which suppresses honey bee immune systems, increasing honey bee susceptibility to other stressors like pathogens and pesticides^13–15^. The role of *Varroa* mites on honey bee colony declines is thus significant and has global implications for *A. mellifera* populations^13,16^.

Honey bees can use social immunity to defend against mites and other pathogens. Social immunity includes behavioral defenses, such as hygienic behavior and grooming^17^. While hygienic behavior is a general response to pathogens and parasites in the colony, *Varroa*-sensitive hygiene consists of bees identifying mite-infected larvae, uncapping the brood cell, and removing the infected larvae from the colony. The latter two behaviors disrupt the *Varroa* life cycle—which depends upon reproduction in larvae^18,19^. Honey bees can also use their legs to groom themselves and other individuals to dislodge *Varroa* mites^20,21^. In addition, honey bees can bite off mite legs to limit mite mobility and parasitization: colonies with higher rates of mite leg mutilation have lower mite infestations^17,22^. Such behavioral defenses are heritable and vary in intensity among different genetic lines^21,23,24^. Feral *A. mellifera* populations are likewise infested with *Varroa* but can evidently survive without receiving miticide treatments^25,26^. The extent to which behavioral social immunity contributed to feral honey bee resilience against *Varroa* is not yet clear.

*Scutellata*-hybrids (“Africanized” bees) are the result of genetic introgression between primarily European honey bee subspecies and *Apis mellifera scutellata*. They have spread from Central and South America into the U.S. as far as 34° N in California^27^. In San Diego, 70% of feral bees have African ancestry^28^. Multiple studies have found that *scutellata*-hybrids remove a greater proportion of infested brood compared to Carnolian, European, and Italian subspecies^21,23,24^. In laboratory self-grooming assays in which researchers placed *V. destructor* mites on honey bees, *scutellata*-hybrids bees self-groomed more rapidly and with greater intensity than Carnolian bees^21^. In addition, *scutellata*-hybrids may have other ways of resisting *Varroa*.

Our goals were therefore to understand the social immunity of feral *scutellata*-hybrids and managed bees and to determine if different behavioral defenses could successfully counter *V. destructor.* Although hygienic behavior and grooming in *scutellata*-hybrids has been studied in Brazil, Central America, and Texas^20,21,24,29^, there are limited studies on these behaviors in southern California, where feral *scutellata*-hybrids are highly abundant and thus highly successful^28^ despite receiving no miticide treatments because they are mostly not managed bees. Even when they are managed, beekeepers report that feral *scutellata-*hybrids do not require treatment against *Varroa*. In fact, based upon studies that quantify floral visitation by a wide range of pollinators, the proportion of floral visits and, thus, the potential abundance of *scutellata*-hybrids relative to all other pollinators is greater in the San Diego region than anywhere else in Central or South America and, for a continental site, is second globally to only one location in Kenya^1^. We therefore compared the behavioral defenses of feral *scutellata*-hybrids and managed *A. mellifera* colonies against *V. destructor* in San Diego. We measured hygienic behavior with pin-kill assays, conducted self-grooming behavioral assays in the lab, and measured mite mutilation by colonies.

## Materials and methods

### Study site and colonies

We conducted our study between January 2021 and April 2022 at two apiaries: the Biology Field Station (BFS) (32.885614137503346, −117.22997498068543) at the University of California San Diego containing only commercial beekeeping stock and, 13.4 km away, the Elliott Chaparral Reserve (ECR) (32.89564249287754, −117.08667665732133) containing only colonies of feral origin. The key goals of our study were to determine if there were differences between (1) mite infestation levels and (2) mite-resistant social immunity behaviors of these two types of bees. We used separate apiaries instead of a common garden design where all colonies are maintained in the same location, despite the latter being a more ideal setup for controlling multiple environmental factors. When feral and managed colonies were placed together, we noticed that feral colonies robbed managed colonies, leading to a high percentage of managed colonies dying out, particularly in October and November. Perhaps the most problematic aspect of robbing, with respect to this study, is that robbing allows mites to spread between colonies^30^. In addition, having mite-infested *scutellata*-hybrids near managed colonies that were regularly inspected and treated against mites could have quickly led to even more severe mite infestations. Thus, we used two separate apiaries: one for *scutellata*-hybrids and one for managed Apis mellifera ligustica colonies.

At the BFS, we used 19 *A. mellifera ligustica* colonies obtained as nuclear colonies from bee breeders in northern California beyond the northernmost zone of *scutellata*-hybrids and requeened, as necessary, with European *A. mellifera ligustica* queens (ancestry tested with molecular methods as described below). We began our study with 15 BFS colonies, but replaced the four that later absconded and therefore used 19 over the course of our experiments. The BFS colonies were managed using best management practices and were regularly treated with acaricides, as needed, when mite levels exceeded 3 mites per 100 bees^31^. Treatments were calibrated to the degree of mite infestation and managed colonies were only treated if they had more than 3 mites/100 bees. No colony received more than three mite treatments during our study. Beginning in February 2021, four colonies were treated with Apivar (3.33% Amitraz) for 56 days. On June 2021, Apiguard (25% thymol) was applied for four weeks to 13 colonies. One colony was treated with Mite-Away Quick Strips (46.7% formic acid) in July 2021. Then, in August 2021, four other colonies were treated with Mite-Away Quick Strips (46.7% formic acid) for 21 days. Apivar was applied to three colonies for 56 days in October 2021 and the same treatment was applied to three other colonies on November 2021. BFS bees were also fed supplemental sucrose solution (50% v/v) and pollen patties (Ultra Bee High Protein Pollen Substitute, 58% crude protein, Mann-Lake Bee & Ag Supply Ltd) *ad libitum* from June through February when floral resources were less available. Managed colonies were also placed on raised stands with mineral oil-filled legs to exclude ants.

We used 15 feral *A. mellifera* colonies at the ECR. These colonies were obtained as rescued feral swarms and feral colonies from different locations around San Diego, California. All of these colonies were verified to be *scutellata*-hybrids via genomic analysis (see below). Like BFS colonies, each ECR colony was housed in a 10-frame Langstroth hive. However, none of the ECR colonies were treated with miticides or other chemicals, and they were also not fed sucrose solution or pollen patties. Feral colonies were not protected from ants. All colonies at both sites had access to water, as required by San Diego County apiary regulations, in horse troughs. We surveyed for mites and measured honey bee colony size roughly once per month at each field site.

### Measuring colony genetic ancestry

*Scutellata*-hybrids have a high degree of ancestry from African (A) honey bee subspecies that can be measured by the A lineage content of their genomes^32^. Zarate et al.^27^ showed that the percentage of A lineage content in *scutellata*-hybrid bees in San Diego, California is, on average 38%. To determine the genetic ancestry of the colonies from two apiaries, we collected three honey bee workers between August 28-November 15, 2021 from ten colonies at the BFS and ten colonies at the ECR by selecting workers from the colonies that we were using. We followed the procedures described by Zarate et al.^27^. In brief, we selected young bees on brood combs to ensure that they were from the hives being studied and not non-nestmates such as robbers. We euthanized the honey bees and preserved them in 100% ethanol at −20 °C. DNA was extracted from the crushed head of each bee using the recommended protocol of the Qiagen DNAeasy Blood & Tissue extraction kit (Catalog ID: 69504). The extracted DNA was submitted for DNA KAPA library construction and whole-genome sequencing at the Institute for Genomic Medicine (IGM), UC San Diego. The sequencing was performed using an Illumina NovaSeqS4 platform to produce 150-bp paired-end reads at ~ 20× coverage. The raw reads generated from sequencing, along with those downloaded from NCBI, were subjected to quality control and length filtering. These reads were then aligned to the reference genome assembled by Wallberg et al.^33^ to estimate ancestry proportions from the A, M, C, or O evolutionary lineages. The program ANGSD v0.930 was used to identify variant sites and estimate genotype likelihoods across 159 honey bee genomes. We estimated honey bee lineage ancestry proportions using NGSadmix software and a panel of 201,975 SNPs with the admixture pipeline described in Zarate et al.^34^. The genetic data on our *scutellata*-hybrid colonies also appears in Zarate et al.^35^, but are summarized here, with attribution, because this data is essential for establishing that our feral colonies are *scutellata*-hybrids.

### Determining bee colony sizes

Colony census surveys were conducted between April 2021 and April 2022, a total of eight times per field site. We based our colony size measurements on standard methods described by Delaplane et al.^36^. Our modified method used the Liebefeld technique described in Dainat et al.^37^, but takes into account the fluctuating bee densities on each frame side, as opposed to using a standardized estimation of 1100 workers per deep Langstroth frame. We took photos of every frame side with bees with an iPhone (iPhone XR and iPhone 13). Using GIMP 2.10 software, 5 × 7 (height × width) grids were electronically overlaid on these pictures to subdivide frames into smaller cells (450 × 450 pixels) for easier measurement. On each side of each frame, we selected a cell with a bee density representative of the majority of the cells in the photo and counted the number of individual bees in that specific cell. On average, this value was 30.57 ± 2.85 (mean ± 1 standard deviation) bees per cell. We then multiplied this number by the number of cells occupied by bees in the frame, repeating the process for each frame side. We then summed our counts from all frames with bees to determine colony size. To validate our method, we also randomly selected some frames, counted all bees from photos, and then applied our estimation method, as described above. These counts were in good agreement. To ensure consistency between researchers counting bees, we randomly selected frame photos and had both researchers count bees on the same photos during count training. We compared the counts made by the trainer and trainee with linear regression, and trainees needed to obtain R^2^ ≥ 0.85 before they were allowed to collect count data used in our analyses.

### Pin-kill assays

We quantified hygienic behavior with a standard pin-kill assay: measuring the degree to which a colony will remove dead larvae from February 2021 to October 2021 (total of 25 assays using 14 managed colonies and 23 feral colonies). We used the pin-kill assay instead of the freeze-kill assay because both assays did not yield significantly different results in *scutellata*-hybrids^38^ and because the pin-kill assay was easier to implement at our more remote ECR apiary. Both freeze-kill and pin-kill assays are used to measure hygienic behavior^13,39,40^. Spivak and Downey^41^ suggest that freeze-kill assays are more effective measures of *Varroa*-sensitive hygiene than pin-kill assays since olfactory signals from pin-killed brood may not reflect those of *Varroa*-parasitized larvae^42^. However, Danka et al.^43^ found no strong link between freeze-kill assays and hygienic responses. Boecking et al.^44^ reported a correlation between removal of pin-killed and *Varroa*-infested larvae, and Shakeel et al. (2020) observed similar hygienic behavior in freeze-kill and pin-kill assays. Direct measures of *Varroa* resistance—inspecting brood for *Varroa*-infested cells^45^ and measuring *Varroa* reproduction^46^—can be used to reliably quantify hygienic behavior, although the former method, if used to compare the resistance of feral vs. managed colonies to *Varroa* infestation, assumes that all colonies at both sites had similar exposure throughout the year to *Varroa*. We therefore used pin-kill assays with the understanding that there are limitations to such assays.

Only colonies with a substantial amount of capped worker brood (at least two full frame sides) were used to ensure that colony fitness was not substantially disrupted by the assay. We did not use sections of comb with drone brood since drone brood was not present at all times and was not available in large patches throughout the year. A circular indentation was made in capped worker brood with a 119 mm diameter metal cylinder (111.2 cm^2^, mean of 260.62 capped brood cells) to mark the assay area. Two indentations were made: with one per treatment. In the pin-kill treatment, all capped brood cells within the circular indentation were perforated with a #2 insect pin^47^ to kill developing larvae. In the control treatment, the circular indentation was left without perforation of capped brood. Photos were taken directly before perforation and 24 h later to measure the number of pin-killed brood removed by the colony from the assay area . To ensure that the natural emergence of adult honey bees was not mistaken for hygienic behavior, we excluded data when there were visual cues of emergence or when control treatments had a removal rate higher than 15%^47^.

### Behavioral assays of mite grooming

Behavioral assays were conducted between February 2022 and April 2022 on 298 bees from 11 managed colonies and 10 feral colonies. Worker bees were collected with individual snap vials (2 cm × 5 cm) from the comb surface. To obtain worker bees at specific ages, it is necessary to paint or mark them as they emerge. However, such marking could affect their self-grooming behavior, and we therefore did not use worker bees of a specific age. Our workers likely represent a broad range of ages in both BFS and ECR colonies. We were, however, able to exclude newly emerged bees, which are easily distinguished by their appearance^48^. Age can affect the speed at which bees react to *Varroa* mites^49^, however, our capture method should have collected, on average, bees of similar ages in each colony at each field site. Collected workers were stored in snap cap vials individually in dark 30℃, 50% relative humidity (RH) incubators until self-grooming trials^50^.

Living *Varroa destructor* mites were collected via the sugar shake method in which bees were dusted with powdered sugar and shaken through a white tulle fabric (as a filter) to dislodge mites^51^ from colonies at the BFS apiary because these colonies had higher mite infestation rates. Mites were collected from powdered sugar debris and placed in a 250 mL glass beaker coated with a Fluon perimeter at the top to prevent mite escape. Beakers with mites were wrapped in perforated plastic wrap and incubated in the dark at 30 °C and 50% RH in preparation for self-grooming assays. Before self-grooming trials, a fine-tip 0000 paint brush was used to wipe off all the remaining powdered sugar from the mites because the ability of *Varroa* to attach to honey bees can be decreased by excessive sugar. Only mites that were in good condition, determined by their ability to climb onto the paint brush were used for trials. A paint brush was used to transfer mites to the Petri dish. We used one mite per bee per trial. Mites were not reused. We compared self-grooming behaviors with a no-*Varroa* control treatment: a paint brush without a mite was briefly placed inside the Petri dish to simulate the mite transfer process.

Self-grooming bioassays were conducted at room temperature (21 °C) in plastic 60 mm × 15 mm Petri dishes lined with press-in beeswax comb foundation. Per trial, two of these Petri dishes were vertically orientated to simulate natural colony comb orientation. A Sony High-Definition Video Camera Recorder (HXR-NX70U) was mounted onto a tripod so that it was level with the Petri dishes. A lamp with a 5 Watt Light Emitting Diode bulb (3000 K color temperature) illuminated the bees. Only bees that were healthy and able to travel vertically up their vial were included as possible participants for these behavioral trials. Once two bees from the same colony were chosen, they were chilled in an ice bucket until their activity slowed (about 1 min) to facilitate transfer into the test dishes and placement of irritants. Any bees that did not quickly recover from this chilling after being placed in a dish were excluded.

The durations of self-grooming behaviors were video recorded for 5 min and a single observer classified these behaviors^20^. The observer was blind to whether bees came from feral or managed colonies. Behavior was categorized as “Weak Cleaning” (legs stroking the head, thorax, or abdomen or metathoracic legs) or “Intense Grooming” (legs stroking multiple body parts simultaneously). For Weak Cleaning, we also noted the body part groomed. We also scored “Attempting to Fly” when a bee buzzed its wings in an attempt to fly and scored “Mite Removal” when bees successfully displaced the mite off their body. The time elapsed before this successful mite removal was also recorded. Different behavioral events were delineated by workers stopping grooming for at least one second or switching to groom a different body part. Lastly, we measured “Grooming Latency,” defined as the time elapsed before the first self-grooming behavior^52^. If bees did not display any self-grooming during the 5-min trial, grooming latency was recorded as 300 s (the entire trial duration). After trials were completed, the beeswax foundation was removed and the Petri dishes were washed and disinfected in a Liquinox detergent and water solution. After drying, new press-in wax foundation was placed in the clean Petri dishes for additional trials.

### Measuring colony mite levels

Mite populations were monitored from April 2021 to April 2022. To measure colony mite levels, we placed sticky traps (Mann Lake #DC-081) under the frames of bee colonies^51^. The bottom board was replaced with a wire mesh through which mites, but not bees, could fall through to a cardboard board sprayed with a thin layer of canola oil as an adhesive^53–55^. Five days after trap placement, we counted all adult female *V. destructor* mites, confirmed by their brown color and appearance^52,54^, that we caught on the trap board. We note that the accuracy of estimate based upon counting mite falls may be affected by increased falls in colonies when treated with miticides. At the BFS apiary, all colonies were placed on ant-excluding legs filled with mineral oil, and thus ants could not remove fallen mites. At the ECR apiary, colonies were not placed on such legs because the goal of this apiary is to eliminate as many protections as possible. It is therefore possible that ECR colonies had somewhat lower mite counts due to ants removing mites. However, whenever we observed ECR colonies to census their size and to collect the mite boards, we found little evidence of ants.

We did not use *Varroa* washes (collecting a given number or volume of bees and washing them with alcohol or soap solution to dislodge mites^51^) because such washes kill the collected bees and some of our feral honey bee colonies had much smaller populations of workers than our managed colonies and were therefore more vulnerable to the loss of workers (see Zenodo repository DOI: 10.5281/zenodo.7927335 for all colony size data).

### Measuring damage to mites

*Varroa destructor* mites were collected with a fine 0000 paint brush over a five-day period from sticky traps under colonies (the same ones used to measure mite colony levels). Mites were then frozen at −20 °C until analysis. For each colony, all collected mites (or the first 50 mites collected, whichever came first) were viewed under a dissecting scope (Nikon C-PS) illuminated with a Dyna Lite 150 Fiber Optic light to measure leg damage. The number of legs removed from each mite, leg location, and the proportion of each leg removed was recorded. Proportional leg damage was measured in increments (0, 0.33, 0.66, or 1) per leg per mite. Immature *V. destructor* (recognized by their yellow color) and empty dorsal shields were excluded from the analysis^52,54^, allowing us to control for mite age and eliminate other potential causes for mite leg damage such as ant predation^56^.

### Statistics

We used Fisher’s Exact test to compare the numbers of feral and managed bee colonies that absconded^57^. All other statistical analyses were performed using JMP Pro v16.0.0 software, and all reported data are presented as mean ± one standard error. Repeated Measures Mixed Models (REML algorithm) were used to analyze all data, with colony treated as a repeated measure and a random variable nested within field site. To investigate changes in colony size over time, we tested the effects of field site and day of year (fixed effects) on colony size. We also tested the effects of field site, day of year, and colony size (all fixed effects) on colony mite infestation rates. Mite infestation rates were log transformed based upon inspection of model residuals.

To determine the A lineage ancestry of colonies from the BFS and ECR, we used a Repeated Measure Mixed Model with field site (a fixed effect) and colony identity nested within field site (a random effect).

To analyze our pin-kill assay data, we used field site, treatment, irritant nested within treatment, assay day of year, mite infestation rate, and colony size (all fixed effects) on larval removal rates from pin-kill assays. To simplify our model and because our goal was to test for differences in the responses of ECR and BFS bees, we analyzed the effects of the control treatment (which resulted in essentially no removal behavior) separately from the experimental treatment.

To analyze our self-grooming data, we ran separate models to test the effects of field site, irritant, and treatment (all fixed effects) on the following durations: durations of weak cleaning, intense cleaning, attempting to fly, time to mite removal, total time grooming, and grooming latency. All duration data were log transformed based upon inspection of model residuals. Tukey Honestly Significant Difference (HSD) tests were used to make corrected all pairwise comparisons between treatments: no mite or live mite.

To analyze mite damage data, we used field site and assay day of year as fixed effects. Mite biting was assessed by calculating the total proportion of legs bitten per mite and percentage of damaged mites. The data were log-transformed based upon inspection of model residuals. We also analyzed the effect of field site, day of year, percentage of damaged mites, and number of legs bitten per mite (all fixed effects) on mite infestation levels per colony to assess the influence of mite biting on mite infestation rates. Additionally, to determine if certain mite legs were more frequently damaged, we tested the effects of field site and Leg ID (both fixed effects) on the proportion of leg bitten on an individual mite level, with mite as a random effect and as a repeated measure nested within colony. Pairwise comparisons for damage among legs were corrected using a Tukey (HSD) test.

For the pin-kill assays, we ran Repeated Measures Mixed Models (REML algorithm) with all interactions. To compare the between control and pin-kill treatments, we ran such a repeated measures model with field site, treatment, month (a continuous variable) and colony nested within field site (random variable). To examine the effects of multiple factors on hygienic behavior, we ran a model with field site, colony nested within field site (random variable), month (a continuous variable), colony size, mite infestation rate (mites/100 bees) and all interactions.

## Results

### Absconding was similar for feral and managed colonies

There was no significant difference in absconding between feral and managed colonies (5 out of 15 feral and 4 out of 15 managed colonies absconded, Fisher’s Exact 2-tailed test, *P* = 1.0).

### Feral colonies were *scutellata*-hybrids

At the ECR, colonies had 41.9 ± 2.7% A group genomic ancestry, but BFS colonies had significantly lower A group genomic ancestry (24.2 ± 10.5%, *F*_1,18_ = 39.32, *P* < 0.0001). Colony identity accounted for 62% of model variance.

### Colony sizes and mite infestations did not significantly change over time

**Figure 1:**
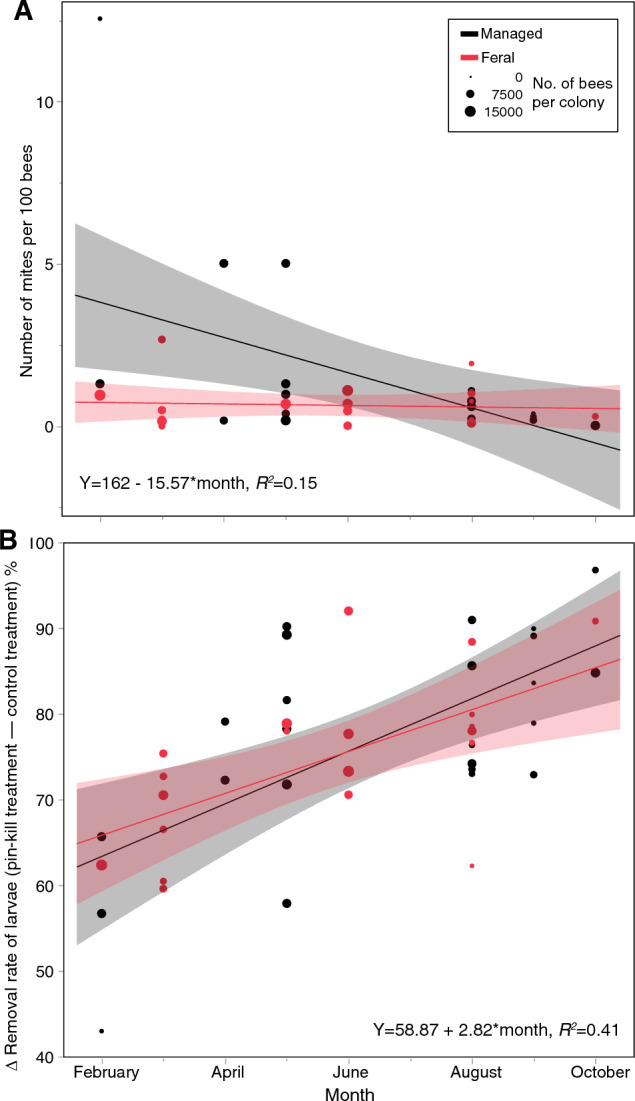
Mite infestation and hygienic behavior in feral (*scutellata*-hybrid) and managed (*A. mellifera ligustica*) colonies over time (February to October 2021). (**A**) Number of mites per 100 bees and (**B**) hygienic behavior as measured from pin kill assays are shown. Removal of control larvae was close to zero at all times, and thus the removal rate and its increase over time reflects changes in hygienic behavior. The linear regression equations are calculated from the data at both field sites because behavior at the two field sites did not significantly differ. Dot diameters reflect colony sizes, and 95% confidence intervals based upon model parameters (see “Statistical methods”) are shown. Colony sizes were only measured from April 2021 to April 2022. Because we found no significant changes in colony sizes over time, the colony sizes in April 2021 were therefore used as the sizes of these colonies in February and March of 2021 for this figure.

Honey bee colony sizes and mite infestation data were collected from April 2021 to April 2022 from 19 BFS colonies and 15 ECR colonies. Colony sizes did not change significantly over time (time: *F*_1,183_ = 2.76, *P* = 0.10) or between field sites (*F*_1,29_ = 0.52, *P* = 0.48), and thus we did not examine the effect of colony size on mite infestation levels. The interaction of date × field site was not significant (*F*_1,182_ = 2.54, *P* = 0.11). Colony accounted for 25% of model variance. Mite infestation rates did not change significantly throughout the year (*F*_1,164_ = 3.04, *P* = 0.08) and did not differ between feral and managed colonies (*F*_1,32_ = 1.20, *P* = 0.28, Fig. 1A).

### Hygienic behavior in *scutellata*-hybrids and managed bees increased over time.

Hygienic behavior was evaluated with 25 pin kill assays using 14 managed colonies and 23 feral colonies between February and October 2021. As expected, pin-kill treatments significantly increased larval removal rate compared to control treatments (*F*_1,67_ = 2650.83, *P* < 0.0001). On average, the pin-kill treatment resulted in the removal of 78.6 ± 1.6% of pin-killed larvae, while the control treatment resulted in the removal of 1.8 ± 0.74% of larvae.

In the full model, no significant interactions were found (*F*_1,13–28_ ≤ 0.51, *P* ≥ 0.48), and there were no significant effects of field site (*F*_1,17_ = 0.18, *P* = 0.67), mite infestation rates (*F*_1,35_ = 2.07, *P* = 0.16), or colony size (*F*_1,36_ = 2.15, *P* = 0.15). However, there was a significant positive effect of month (*F*_1,37_ = 17.62, *P* = 0.0002), with removal rates increasing over time and as colonies grew. On average, removal rates (hygienic behavior) increased by 2.8% per month during the duration of this hygienic behavior experiment (Fig. 1B).

### Some mite grooming behaviors changed over time, but grooming did not differ between *scutellata-*hybrids and managed bees

Between September 2021 and April 2022, we conducted mite grooming behavior tests on 298 bees from 11 managed colonies and 10 feral colonies. When we compared the effects of irritant (mite vs. no mite) on bee behaviors for all time points, we found no effects of field site (*F*_1, 8_ ≤ 1.59, *P* ≥ 0.24) or the interaction field site x mite (*F*_1, 137_ ≤ 2.78, *P* ≥ 0.09). However, the presence of a mite on a bee increased the number of times that a bee weakly cleaned (*F*_1, 138_ = 6.43, *P* = 0.01) or strongly cleaned (*F*_1, 138_ = 4.60, *P* = 0.03) itself and the total time that bee spent grooming (*F*_1, 138_ = 8.18, *P* = 0.005).

**Figure 2:**
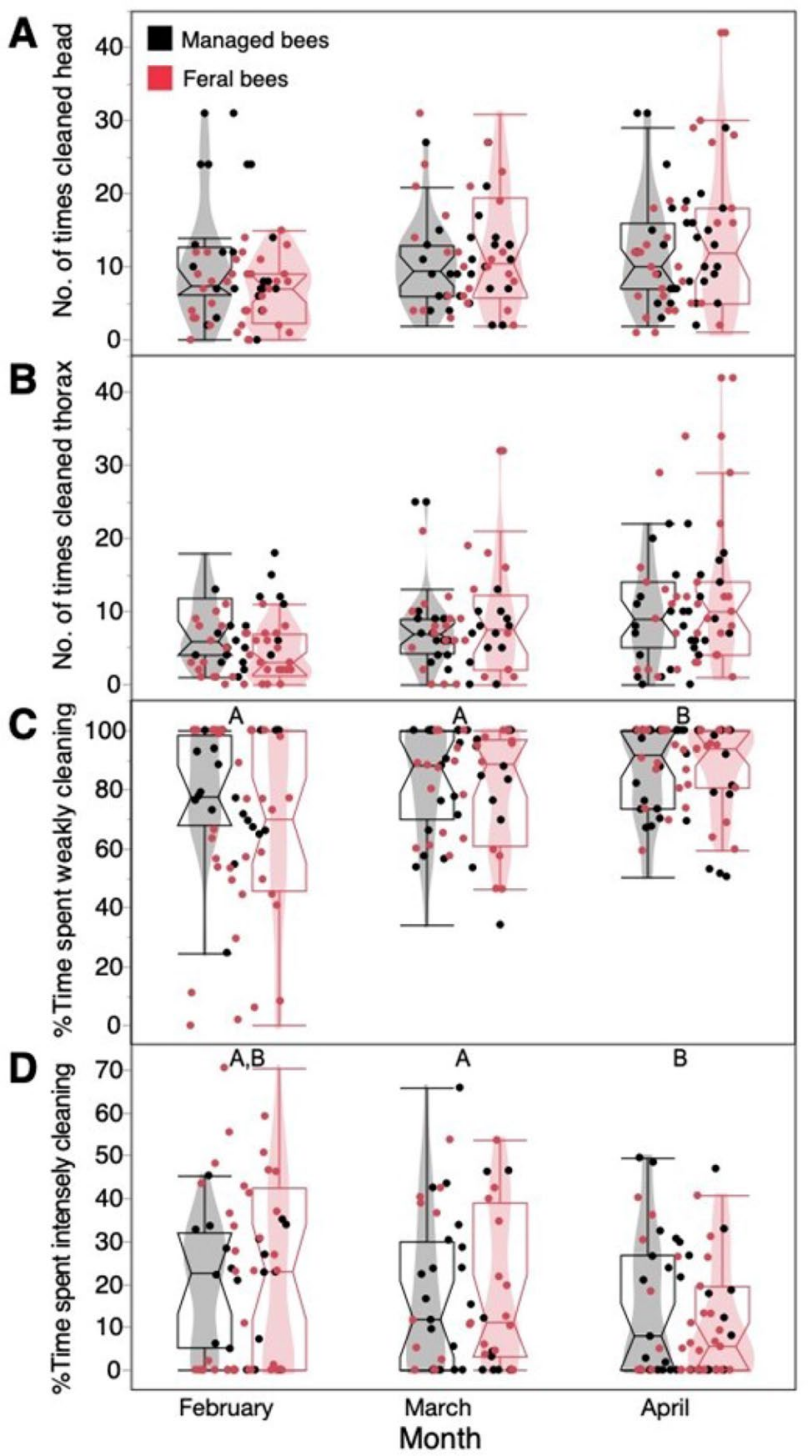
There were no significant behavioral differences between feral (*scutellata*-hybrid) and managed (*A. mellifera ligustica*) bees in any assay measure within any month (*P* > 0.05). We show the typical results for representative behaviors: the number of times bees with mites cleaned their (**A**) head or (**B**) thorax. However, the percentage of time that bees with mites spent (**C**) weakly cleaning slightly and significantly increased over time (*P* = 0.027). (**D**) The percentage of time that bees with mites spent intensely cleaning slightly decreased over time (*P* = 0.029), mainly between April and March. Box plots overlaid with violin plots and all data points are shown. Different letters indicate differences between behaviors by month (Tukey HSD tests, *P* < 0.05).

We then examined the behaviors of bees with mites over time. There were no effects of field site on the grooming behavior of the honey bees (*F*_1, 7_ ≤ 2.38, *P* ≥ 0.16) and no significant interaction between field site and day of year (*F*_1,6_ ≤ 3.53 *P* ≥ 0.11). Only three variables were significantly impacted by day of year: the number of times bees cleaned their thorax (*F*_1,6_ = 6.20, *P* = 0.049), percentage of time spent weak cleaning (*F*_1,6_ = 12.89, *P* = 0.01, and percentage of time spent intense cleaning (*F*_1,6_ = 13.79, *P* = 0.0089). When we analyzed this data by month, bees spent a greater percentage of time weakly cleaning (*F*_2,6_ = 7.36, *P* = 0.027) and a smaller percentage of time intensely cleaning themselves (*F*_2,5_ = 7.32, *P* = 0.029) in later months (Fig. 2). No other measures had measurable differences over time (*F*_1, 7_ ≤ 3.24, *P* ≥ 0.12).

### Damage to mites from both bee types was higher on their chemosensory forelegs

We examined 4,161 mites collected from honey bee mite traps (*N* = 1,643 mites from 20 BFS colonies and *N* = 2,518 mites from 15 ECR colonies) between January 2021 and April 2022 to evaluate mite biting behavior. We found similar levels of damage to mites in both feral and managed colonies, and this damage did not change with time of year or the level of mite infestation. In the full model (*R*^2^ = 0.06) for the mean proportion of legs bitten per mite, field site (*F*_1, 35_ = 1.22, *P* = 0.28), day of year (*F*_1,179_ = 0.07, *P* = 0.79), and mites per colony (*F*_1,191_ = 0.14, *P* = 0.71) were not significant, and there were no significant effects of any interaction (*F*_*1,190*_ ≤ 1.89, *P* ≥ 0.17).

**Figure 3:**
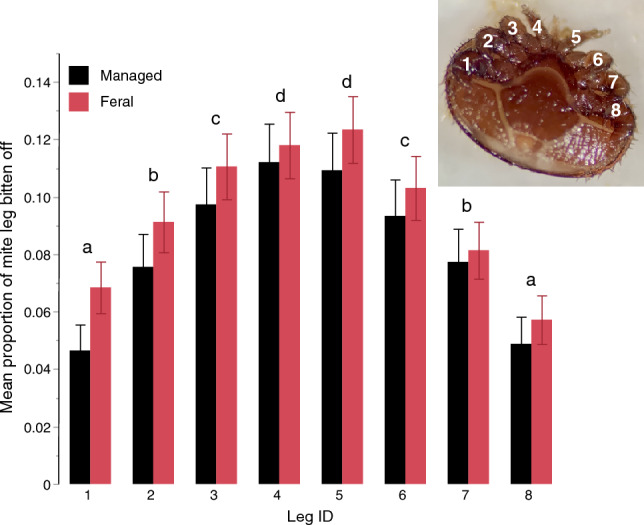
Anterior mite legs were bitten to a greater degree than posterior legs in mites collected in feral (*scutellata*-hybrid) and managed (*A. mellifera ligustica*) colonies. Inset image shows a mite with numbered legs. Error bars show 95% confidence intervals, and different letters indicate significant differences (Tukey HSD test, *P* < 0.05).

We next focused on how much of each leg was bitten off. There was a significant effect of leg ID (*F*_7,28,889_ = 103.64, *P* < 0.0001) because a greater proportion of anterior legs were bitten as compared to posterior legs (Tukey HSD test, *P* < 0.05, *R*^2^ = 0.70, Fig. 3). However, there were no significant effects of bee type (*F*_1,29_ = 0.13, *P* = 0.72) or the interaction leg ID x field site (*F*_7,192_ = 0.61, *P* = 0.75). We therefore combined the data from feral and managed colonies and calculated that, on average, 12% of the length of the two anterior forelegs (legs 4 and 5), which house the chemosensory pit organs, were bitten by bees on average, but only 5–6% of the length of the posterior legs (legs 1 and 8) were bitten off by bees (Fig. 3). Thus, bees shortened the mite legs with chemosensory organs to a 2.2-fold greater degree than the posterior legs without these chemosensory organs.

## Discussion

Despite not receiving any treatments against mites, feral *scutellata*-hybrid colonies had similar mite infestation levels compared to miticide-treated, managed *Apis mellifera ligustica* colonies. These two types of bees also had similar levels of hygienic and grooming behaviors. This unexpected result suggests that feral colonies might be controlling mite levels through mechanisms beyond the social behaviors we examined (hygienic removal, self-grooming, and mite biting). Over time, mite infestation levels (mites per 100 bees) in both bee colony types remained steady, while hygienic behavior and some grooming behaviors increased, including a 2.8% per month rise in the removal of pin-killed larvae. If these small increases in hygienic behavior, based upon the pin-kill assay, helped to reduce the mite population, they did not have a strong effect on overall mite numbers. Without external interventions or colony resistance mechanisms, *Varroa* mite populations can grow exponentially^58^. For the managed honey bee colonies, the relatively steady level of mite infestations therefore likely reflects the multiple miticide treatments given throughout the year in response to mite measurements. For the feral *scutellata*-hybrid colonies, one might expect seasonal increases in mite levels, such as those observed by Mondragón et al.^22^ in Mexico. We note that *scutellata*-hybrids may be more abundant in the San Diego region than any other region in Central or South America in which floral visitation by a wide range of pollinators has been studied^1^, suggesting that these successful feral bees may have adaptations to control *Varroa* infestations. Perhaps the most intriguing finding was that both managed and feral bees bit off mite chemosensory forelegs, vital for mite reproduction, at a 2.2-fold greater rate than other legs. This action could potentially interfere with the mites' capacity to locate and parasitize brood cells.

*Varroa* resistance in *A. mellifera* and *Apis cerana*, the original host of *V. destructor*, appears to arise from an array of behavioral strategies and traits^59–61^. For example, swarming or absconding leads to a period of broodless that significantly reduces mite infestation levels^62^. The relative value of these traits and the potential for synergistic interactions among these traits remains to be fully elucidated. However, we know that some honey bee brood can reduce mite reproduction^63^. In the widely studied *Varroa*-sensitive hygiene (VSH) behavioral syndrome, workers selectively remove *Varroa*-infested larvae^61^. Workers can also recap brood cells to hinder *Varroa* development and reduce mite fecundity. Colonies that are resistant to mites have increased rates of recapping *Varroa*-infested brood cells in comparison to mite-susceptible and mite-naïve colonies. This recapping behavior correlates with the hygienic removal of *Varroa*-infested brood and higher mite infertility^19^. Oddie et al.^64^ compared the hygienic and grooming behaviors (measured as the proportion of damaged mites per colony) and *Varroa*-sensitive hygienic behavior (VSH) or the removal of larvae infested with *Varroa*. They found that grooming and *Varroa*-sensitive hygienic behavior did not significantly differ between colonies naturally selected to be more *Varroa*-resistant and colonies that were *Varroa*-susceptible, although mite reproductive success was reduced in *Varroa*-resistant colonies. It would be useful to conduct additional measurements of different VSH behaviors within the same genetic stocks. Spivak and Danka^65^ reviewed the literature on hygienic behavior and suggested that the hygienic removal of freeze- or pin-killed larvae may not correlate with a colony’s VSH, but serve rather as a screening tool for a colony’s VSH potential. In general, the genetics underlying VSH and other *Varroa*-resistance traits are complex^60^.

The relatively high lineage A ancestry (24.2 ± 10.5%) of our managed honey bees is a limitation of our study. Using managed honey bees with significantly lower lineage A ancestry could yield different results, and we suggest that routinely analyzing and including the detailed genomic ancestry of bees used in *Varroa*-resistance studies could be useful for testing the hypothesis that there is a positive correlation between the proportion of lineage A ancestry and *Varroa*-resistance. However, our *scutellata*-hybrids had 1.7-fold greater lineage A ancestry than our managed bees, and yet we found no difference in hygienic behavior. Anothger limitation of our study arose from housing managed colonies at one apiary and *scutellata*-hybrid colonies at a different apiary to avert robbing, which might exacerbate *Varroa* mite transmission, and to eliminate mite transfer between our managed and *scutellata*-hybrid colonies. Because we found no significant differences in mite infestation levels and *Varroa*-resistant behaviors between these sites, potential differences arising from the different apiary sites did not seem to influence our results, although this remains a possibility. Both apiary sites were immediately adjacent to areas with abundant feral colonies that could have been a source for *Varroa* mites. The similar rates of mite infestation that we found in our *scutellata*-hybrids and managed honey bees are consistent with the findings of Mondragon et al. (2005) who also found no significant difference in the mite infestation rates of *scutellata*-hybrids, European-hygienic x *scutellata*-hybrid crosses, or European-commercial x *scutellata*-hybrid crosses in Mexico. With respect to seasonality, there were no changes in mite infestation levels over time in our study. However, some studies in different regions have shown that mite infestations increase in late summer and fall as compared to spring^66,67^. These studies were conducted in Denmark and Germany, where winter completely interrupts brood production and therefore mite reproduction. In a warmer climate such as that of San Diego, brood production and the associated need for pollen foraging occurs year-round^68^, although how these climate differences translate into a steady level of *Varroa* parasitization in feral colonies is unclear. However, in Mexico, Mondragon et al.^22^ found a decline in mite infestation levels from February to June and August, which they attributed to increased levels of colony strength during nectar flow^69^. If *Varroa*-resistance is associated with the percentage of *scutellata* genes in *scutellata* hybrids (although this remains to be clearly demonstrated), it is relevant to note that workers from our feral colonies contained, on average, almost half as much African (Lineage A) genomic DNA as compared to Mexican *scutellata*-hybrid workers (41.9% for our San Diego bees and 76.7% for Mexican bees)^34^.

### Hygienic removal of pin-killed larvae

Throughout our year-long study, we observed a slight increase in hygienic removal of pin-killed larvae in both *scutellata*-hybrid and *A. mellifera ligustica* colonies. Despite this increased removal behavior, mite levels remained statistically unchanged, suggesting that this increased hygienic behavior did not reduce, mite populations, although it may have kept *Varroa* levels from increasing. We also found no difference between the hygienic removal behavior of *scutellata*-hybrid and *A. mellifera ligustica* colonies. Previous studies have reported that *scutellata*-hybrids in Mexico and Brazil remove a larger proportion of *Varroa*-infested brood compared to European honey bees: see thesis and conference proceeding citations in^70^. The mean genomic African ancestry of *scutellata*-hybrids is 76.7% in Mexico and 89.6% in Panama^34^. In our study, the lower mean African ancestry (41.9%) of *scutellata*-hybrids may have contributed to reduced hygienic behavior, making them more comparable to our managed bees. However, we recognize that the percentage of African ancestry might not necessarily correlate with hygienic behavior. Given that our *scutellata*-hybrids were all feral, they are likely under strong selection for hygienic behavior. Feral honey bee populations outside the range of *A. mellifera scutellata* hybridization have shown natural selection for mite-control behaviors^71^ and can be selected for traits that reduce mite reproductive success^64^. Overall, our *scutellata*-hybrid colonies removed 76% of pin-killed brood, while managed colonies removed 80%, which falls within the broad range of brood removal levels reported in similar experiments. Palacio et al.^72^ found that hygienic colonies removed 99% of pin-killed brood, while non-hygienic colonies removed 53%.

### Self-grooming behavior

In our self-grooming assays, bees with mites spent more time grooming themselves than bees without mites, as expected, but we found no significant differences between *scutellata*-hybrid and *A. mellifera ligustica* bee self-grooming in response to *V. destructor*. These results are consistent with Kruitwagen et al.^73^, who reported that naturally mite-resistant colonies exhibited similar grooming behavior as managed colonies treated with miticides. The authors suggested that mite-resistant colonies might employ alternative strategies, such as reducing mite fertility or exhibiting higher hygienic behavior, rather than relying on grooming alone^73^. While other studies have shown that *scutellata*-hybrid bees self-groom more than European bees^53,74^, these studies were conducted in Central and South America, where honey bees have higher African ancestry than those in San Diego^34^.

We observed slight increases in the percentage of time that bees with mites spent weakly grooming themselves over time. However, these changes did not impact the mite infestation rate or correlate with the percentage of damaged mites collected. Consequently, it is unlikely that these behaviors contributed to increased mite resistance. The reason for the observed decrease in the percentage of time spent intensely grooming in April as compared to March remains unclear. Potentially, grooming behavior may not be as crucial in colony defense against *Varroa destructor* as previously thought. *Apis cerana*, the original host of *Varroa destructor, Apis cerana,* was reported to intensely groom in response to contact for this mite, unlike the lesser responses of *Apis mellifera*, and grooming was therefore thought to play a critical role in mite-resistance. The role of grooming, brood removal behavior, and mite infertility in the ability of *A. cerana* to resist *Varroa* remains unclear^75^ and much therefore remains to be learned about *Varroa* resistance mechanisms in *A. mellifera*.

### Damage to mites caused by bee biting

We found no significant differences in damage inflicted upon mites by *scutellata*-hybrid and *A. mellifera ligustica* colonies, which is in line with previous findings that showed similar levels of damage between feral and European-African hybrids as well as untreated and miticide-treated colonies^22,73^. In Mexico, *scutellata*-hybrid bees exhibited higher rates of mite mutilation compared to their European counterparts^24^. The lower proportion of *scutellata*-hybrid nuclear genes in feral bees in San Diego may account for these discrepancies. However, in both bee types, mite chemosensory forelegs were bitten off to a greater extent than other legs. These forelegs contain tarsal pit organs that assist mites in locating bee larvae^76^. Research demonstrates that covering these pit organs with nail polish to eliminate chemosensation significantly impaired the mites' ability to find hosts, feed on bee larvae, and reproduce^76^. Therefore, selective shortening or biting of these chemosensory forelegs could help the colony reduce mite infestation, a hypothesis that should be tested in future experiments. However, it remains unclear whether bees specifically target the forelegs or if these legs are simply more vulnerable due to their exposure when mites extend them to detect odors, such as the scent of brood^76^.

## Summary

Without miticide treatments, we expected mite levels in *scutellata*-hybrid colonies to be much higher than in our constantly treated managed colonies. Our findings thus suggest that hygienic behavior, self-grooming, and mite biting, as social immunity behaviors, are unlikely the sole explanation for the relative resistance of *scutellata*-hybrids to *Varroa destructor* in Southern California, an area with a very high abundance of feral *scutellata*-hybrids^1,28^. *Scutellata*-hybrids colonies could employ additional strategies, such as frequent swarming or absconding, which could lead to periods of broodlessness, hindering *Varroa* development due to the mites' reliance on larval hosts. Loftus et al.^62^ showed that swarming colonies had lower mite infestation levels and reduced deformed wing virus titers. However, in our study, we did not find differences in the swarming or absconding of managed vs. feral colonies. We found that 33% of *scutellata*-hybrid colonies and 27% of managed *A. mellifera ligustica* colonies absconded, a non-significant difference. Future research should therefore explore other mechanisms, such as ways in which colonies can decrease *Varroa* reproductive success. Moro et al.^61^ conducted a detailed study that measured worker brood cell recapping, worker *Varroa* sensitive hygienic behaviors, and mite reproduction and found that honey bee colonies are able to resist the mites by mechanisms as yet unknown—an intriguing prospect for future studies on the superabundant *scutellata*-hybrids in Southern California.

## Data Availability

The datasets generated and analysed in the current study are available in the Zenodo repository (DOI: 10.5281/zenodo.7927335).
